# HLA Alleles Influence the Clinical Signature of Amoxicillin-Clavulanate Hepatotoxicity

**DOI:** 10.1371/journal.pone.0068111

**Published:** 2013-07-09

**Authors:** Camilla Stephens, Miguel-Ángel López-Nevot, Francisco Ruiz-Cabello, Eugenia Ulzurrun, Germán Soriano, Manuel Romero-Gómez, Antonia Moreno-Casares, M. Isabel Lucena, Raúl J. Andrade

**Affiliations:** 1 Unidad de Gestión Clínica de Enfermedades Digestivas, Servicio de Farmacología Clínica, Hospital Universitario Virgen de la Victoria, Instituto de Investigación Biomédica de Málaga-IBIMA, Universidad de Málaga, Málaga, Spain; 2 Departamento de Bioquímica y Biología Molecular III/Inmunología, Hospital Universitario Virgen de las Nieves, Universidad de Granada, Granada, Spain; 3 Servicio de Gastroenterología, Hospital de la Santa Creu i Sant Pau, Universitat Autònoma de Barcelona, Barcelona, Spain; 4 Unidad de Gestión Clínica de Enfermedades Digestivas, Hospital Universitario de Valme, Universidad de Sevilla, Andalucía Tech, Sevilla, Spain; 5 Centro de Investigación Biomédica en Red de Enfermedades Hepáticas y Digestivas (CIBERehd), Barcelona, Spain; 6 Red Genómica del Cáncer, Madrid, Spain; University of Navarra School of Medicine and Center for Applied Medical Research (CIMA), Spain

## Abstract

**Background and Aim:**

The genotype-phenotype interaction in drug-induced liver injury (DILI) is a subject of growing interest. Previous studies have linked amoxicillin-clavulanate (AC) hepatotoxicity susceptibility to specific HLA alleles. In this study we aimed to examine potential associations between HLA class I and II alleles and AC DILI with regards to phenotypic characteristics, severity and time to onset in Spanish AC hepatotoxicity cases.

**Methods:**

High resolution genotyping of HLA loci A, B, C, DRB1 and DQB1 was performed in 75 AC DILI cases and 885 controls.

**Results:**

The distributions of class I alleles A*3002 (*P/Pc* = 2.6E-6/5E-5, OR 6.7) and B*1801 (*P/Pc* = 0.008/0.22, OR 2.9) were more frequently found in hepatocellular injury cases compared to controls. In addition, the presence of the class II allele combination DRB1*1501-DQB1*0602 (*P/Pc* = 5.1E-4/0.014, OR 3.0) was significantly increased in cholestatic/mixed cases. The A*3002 and/or B*1801 carriers were found to be younger (54 vs 65 years, *P* = 0.019) and were more frequently hospitalized than the DRB1*1501-DQB1*0602 carriers. No additional alleles outside those associated with liver injury patterns were found to affect potential severity as measured by Hy’s Law criteria. The phenotype frequencies of B*1801 (*P/Pc* = 0.015/0.42, OR 5.2) and DRB1*0301-DQB1*0201 (*P/Pc* = 0.0026/0.07, OR 15) were increased in AC DILI cases with delayed onset compared to those corresponding to patients without delayed onset, while the opposite applied to DRB1*1302-DQB1*0604 (*P/Pc* = 0.005/0.13, OR 0.07).

**Conclusions:**

HLA class I and II alleles influence the AC DILI signature with regards to phenotypic expression, latency presentation and severity in Spanish patients.

## Introduction

Amoxicillin-clavulanate (AC) was marketed over 30 years ago and is today one of the most commonly prescribed antibiotics in Spain and Europe [1], [2]. Its widespread use stems from the effective combination of amoxicillin and clavulanic acid, which extends the antimicrobial spectrum by providing activity against beta-lactamase producing bacteria. Despite being a relatively safe medication, AC has been associated with idiosyncratic drug-induced liver injury (DILI). It is currently the main causative drug in two large DILI registries as well as being associated with the highest hospitalization rate among DILI cases in the Spanish DILI registry [3], [4].

In spite of increasing attention on the impact and pathology of DILI during the last decade, the underlying mechanism of AC hepatotoxicity remains undefined. The clavulanic acid constituent is, however, believed to increase the DILI risk as considerably less hepatotoxicity cases due to amoxicillin alone have been reported [5]. The involvement of immune related components in AC DILI was first suggested in two independent studies conducted in the UK and Belgium. Both studies demonstrated enhanced frequency of the human leukocyte antigen (HLA) class II alleles DRB1*1501 and DQB1*0602 in AC DILI cases [6], [7]. The result was later confirmed in a recent collaborative genome-wide association (GWA) study involving 201 AC DILI cases from the UK, US and Spain [8]. This study also demonstrated variations in HLA allele associations between the Spanish and the British/American cases, suggesting the presence of ethnically different risk alleles in AC-DILI. Such differential allele associations may lead to clinical phenotype discrepancies. For example, AC hepatotoxicity generally manifests as cholestatic or mixed damage, though a comparison of clinical profiles in AC DILI cases of different ethnic origin has demonstrated differences in phenotypic expression, with Spanish cases exhibiting a higher prevalence of developing hepatocellular type of injury [9].

The time frame between starting an AC treatment and the onset of hepatotoxicity can also vary. It is not uncommon to find symptoms of AC DILI commencing after the treatment is completed. In fact, delayed (after treatment cessation) onset is often considered a characteristic feature of AC DILI [9]. The determinant behind a drug’s hepatotoxicity ‘signature’, (consistent clinical and pathological features, latency presentation and severity) is currently unknown, but could to some extent be genetically controlled.

This study was undertaken to further explore differential HLA associations in AC DILI cases recently encountered in a multinational study. Here we have focused on an extended Spanish AC DILI cohort in an attempt to define associations between HLA class I and II alleles and the AC DILI signature with regards to phenotypic characteristics, onset and severity.

## Patients and Methods

### Subjects and Study Protocol

Cases of AC-induced liver injury were selected from those submitted to the Spanish DILI Registry, a collaborative network established in 1994 to prospectively identify DILI cases in a standardized manner. Forty-nine of the cases were included in a recent international study [8] while the remaining 26 cases have not previously been analysed for HLA associations. The criteria for DILI were: an increase in alanine aminotransferase (ALT) ≥3 times the upper limit of normal (xULN) or ≥2 xULN of alkaline phosphatase (ALP) or total bilirubin (TB) ≥2 xULN if associated with elevations of ALT or ALP. The pattern of liver injury was classified based on R value calculations ((ALT value/ULN)/(ALP value/ULN)) as described earlier [10]. An R value of ≥5, 2<R>5 and ≤2 indicates hepatocellular, mixed and cholestatic liver injury, respectively. Liver tests used for the classification of liver damage were the first blood test available after liver injury detection. As a marker of severe DILI, cases were assessed for fulfilling Hy’s Law. Criteria used to define Hy’s Law cases were: hepatocellular injury (R ≥5) with total bilirubin >2 xULN in the absence of other explanations for the combined increase in bilirubin and transaminase values [11]. All submitted cases were evaluated for causality assessment initially by a group of three experts and later through the application of the Council for International Organizations of Medical Science (CIOMS) scale [12]. A detailed description of the operational structure of the registry, data recording and case ascertainment has been reported elsewhere [13].

As a control group for the HLA allele analyses, we selected 885 unrelated bone marrow donors of Caucasian descent, with no previously known history of DILI, from the Immunology Unit at the Hospital Virgen de las Nieves in Granada, Spain. Due to the frequent use of AC in the general population in Spain it was assumed that a large proportion of the controls would have taken AC during some stage of their lives [1].

### Ethics Statement

The study protocol was approved by the local ethics committee of the coordinating center at the Hospital Universitario Virgen de la Victoria in Málaga, Spain. All subjects who took part in the study gave written informed consent, which conformed to the current Helsinki Declaration.

### DNA Extraction and Determination of HLA Class I and II Genotypes

Venous blood was obtained from each subject and DNA was extracted using QIAamp DNA Blood Mini Kit (QIAGEN, Hilden, Germany) according to the manufacturer’s instructions. Genotyping of the HLA class I (A, B and C) and II (DRB1 and DQB1) loci was performed using the LABType® SSO typing test (One Lambda, Canoga Park, CA, USA). Target DNA was amplified by PCR using sequence-specific primers, followed by hybridization with allele-specific oligodeoxynucleotides coupled with fluorescent phycoerythrin-labeled microspheres. The fluorescence intensity was determined with a Bio-Plex 200 system (Luminex xMAP, Austin, TX, USA). HLA alleles were assigned using the HLA-Tools software (One Lambda, Canoga Park, CA, USA).

### Statistical Analysis

HLA allele distributions were compared between DILI patients and controls using a comparison of proportions test, a derivative of the Chi-square test. Means were compared by Student *t* test for independent samples. Analysis of variance (ANOVA) was used for comparison of groups. Where variables did not follow a normal distribution, a nonparametric analysis with the Kruskal-Wallis test was performed. Odds ratios (OR) and 95% confidence intervals (CI) were calculated to assess the relative disease risk conferred by a specific allele. Analyses were performed using the SPSS 19.0 statistical software package program (SPSS Inc, Chicago, IL, USA) and *P*<0.05 was considered to be statistically significant.

To account for the problem of significant associations arising by chance after multiple comparisons, the Bonferroni correction for multiple tests was applied by multiplying the probability value (*P*) by the total number of alleles compared for each locus (n = 19, 27, 19, 27 and 14 for HLA-A, -B, -C, -DRB1 and –DQB1, respectively) to give a corrected *P* value (*Pc*). To correct for HLA class II allele combinations the *P* value was multiplied by the number of DRB1 alleles encountered in this study (n = 27). HLA class I allele combinations were corrected for by multiplying with the total number of alleles in the most diverse locus, ie HLA-B (n = 27).

### Amino Acid Analysis

Evaluation of the HLA alleles (loci A, B, C, DRB1 and DQB1) at the amino acid level, in particular amino acids situated in the antigen binding pockets, and their associations and interactions were conducted using the SKDM software program [14].

## Results

### Description of DILI Patients

Seventy five AC-induced liver injury patients, 46 male/29 female (mean age, 58 years) were analysed. The type of liver damage was classified as hepatocellular (n = 28), cholestatic (n = 22) or mixed (n = 25) with jaundice detected in 88% of the patients. Nineteen patients (25%) fulfilled Hy’s Law criteria. There was a favourable clinical outcome in 73 patients, while two patients developed fulminant hepatic failure.

### HLA Class I and II Genotyping

High resolution genotyping of the HLA class I (A, B and C) and II (DRB1 and DQB1) loci was performed in 75 DILI cases, classified by the type of liver damage (hepatocellular or cholestatic/mixed), and 885 controls. The class I alleles A*3002 (OR = 6.7; CI = 2.8–15.9, *P*/*Pc* = 2.6E-6/5E-5) and B*1801 (OR = 2.9; CI = 1.3–6.2, *P*/*Pc* = 0.0082/0.22) were significantly more common in hepatocellular cases than in the controls, though the latter did not reach significance after Bonferroni’s correction. The association with hepatocellular injury was also extended to the A*3002-B*1801 allele combination (OR = 5.3; CI = 1.9–14.7, *P*/*Pc* = 9.5E-4/0.026). In contrast, the class II allele combination DRB1*1501-DQB1*0602 (OR = 3.0; CI = 1.6–5.5; *P*/*Pc* = 5.1E-4/0.014) was significantly more common in DILI cases with cholestatic/mixed type of damage compared to the controls (Table 1).

**Table 1:** HLA allele distribution in amoxicillin-clavulante induced liver injury patients and controls.

HLA Alleles	DILI (%) n = 75	Hep (%) n = 28	Chol/Mix (%) n = 47	Control (%) n = 885	*P*/*Pc* (OR)
A*3002	11 (15)	8 (29)	3 (6)	50 (6)	^H^2.6E-6/5E-5 (6.7)
B*1801	18 (24)	11 (39)	7 (15)	163 (18)	^H^0.0082/0.22 (2.9)
DRB1*1501	23 (31)	6 (21)	17 (36)	158 (18)	^C/M^0.0024/0.06 (2.6)
DQB1*0602	23 (31)	6 (21)	17 (36)	150 (17)	^C/M^0.0012/0.03 (2.8)

H: hepatocellular type of damage, C/M: cholestatic/mixed type of damage, OR: odds ratio.

### HLA Allele Associations with AC DILI Phenotypes

Clinical data was compared between cases containing the alleles A*3002 and/or B*1801 (referred to as group 1) and those without these two alleles but containing the DRB1*1501-DQB1*0602 allele combination (group 2). The patients in group 1 were significantly younger (54 vs 65 years (*P* = 0.019), 46% vs 83% being ≥60 years of age (*P* = 0.013)). The distribution of males and females also varied between the two groups with a preponderance of males in group 1 (71% vs 29%) and females in group 2 (61% vs 39%) (*P* = 0.038). In terms of liver damage, group 1 had a predominance of hepatocellular type of injury, while cholestatic/mixed type of injury was significantly more common among the patients in group 2. The predominance of hepatocellular injury in group 1 was also reflected in the significantly higher ALT mean value for the same group (*P* = 0.007). The two fulminant liver failure cases fell into group 1, which also had a higher proportion of hospitalizations (71% vs 44%) (Table 2).

**Table 2:** Comparison of demographics, clinical and laboratory findings in amoxicillin-clavulanate DILI patients classified by HLA alleles.

	A*3002 and/or B*1801 n = 24	DRB1*1501- DQB1*0602 (butnot B*1801 or A*3002) n = 18	*P* value
***Characteristics of patients***			
Mean age (range), years	54 (25–88)	65 (35–80)	**0.019**
≥60 years, n (%)	11 (46)	15 (83)	**0.013**
Sex (male/female)	17/7	7/11	**0.038**
Time to onset, mean ± SD, days	17±12	17±14	ns
Duration of treatment, mean ± SD, days	9±6	11±7	ns
Delay onset, mean ± SD, days (number of cases,%)	10±8 (19, 79)	11±12 (11, 61)	ns
***Clinical Presentation, n (%)***			
Jaundice	21 (88)	15 (83)	ns
Hospitalization	17 (71)	8 (44)	ns
*Type of damage*			
Hepatocellular	14 (58)	2 (11)	**0.002**
Cholestatic/Mixed	10 (42)	16 (89)	**0.002**
***Laboratory parameters, (mean ± SD)***			
Total bilirubin, mg/dL	10.3±7.3	9.1±5.8	ns
ALT, xULN	19.4±22.1	5.7±4.7	**0.007**
ALP, xULN	2.2±2.2	2.6±2.3	ns
***Clinical outcome***			
Severe damage (fulminant damage/transplantation), n	2	0	na

ALT: alanine transaminase, ALP: alkaline phosphatase, ULN: upper limit of normal, ns: not significant, na: not applicable.

### HLA Allele Associations with Hy’s Law Cases

In search for HLA alleles associated with severe hepatotoxicity, all cases complying with Hy’s Law criteria (hepatocellular cases (R ≥5) with total bilirubin values >2 xULN) were selected and analysed. To determine the best definition of Hy’s Law criteria we examined the proportion of hepatocellular and cholestatic/mixed cases that were included when considering ‘hepatocellular injury’ to indicate >3, ≥5 and ≥10 xULN of ALT or R ≥5, in conjunction with a total bilirubin value of >2 xULN. With an ALT value >3 xULN, 47 cases qualified of which 60% were cholestatic or mixed cases. The corresponding numbers for ALT ≥5 and ≥10 xULN were 37 cases (49% chol/mix) and 19 cases (26% chol/mix), respectively. When considering an R value ≥5 and total bilirubin >2 xULN 19 hepatocellular cases qualified, of which only three had 2<ALP<4 xULN. Hence, these 19 cases were selected as Hy’s Law cases.

The HLA-A*3002 allele was found to be significantly more frequent in the Hy’s Law cases compared to the controls (OR = 6.0; CI = 2.1–17.2, *P*/*Pc* = 4.9E-4/0.0093). A similar trend was noted for the B*1801 allele, though it did not reach significance (OR = 2.6; CI = 1.0–6.7, *P*/*Pc* = 0.059/1). When comparing hepatocellular cases with total bilirubin >2 xULN with those having <2 xULN, the principal difference noted was the allele combination DRB1*1502-DQB1*0602 being substantially less common in the former group (OR = 0.07; CI = 0.006–0.8, *P*/*Pc* = 0.024/0.64) (Table 3).

**Table 3:** HLA allele distribution in hepatocellular amoxicillin-clavulante induced liver injury patients classified by concomitant total bilirubin values and controls.

	Hep+TB >2 xULN n = 19	Hep+TB <2 xULN n = 9	Controls n = 885	*P*/*Pc* (OR)
A*3002	5 (26%)	3 (33%)	50 (6%)	**4.9E-4/0.0093** § (6.0)
B*1801	7 (37%)	4 (44%)	163 (18%)	0.059/1§
DRB1*1502-DQB1*0602	1 (5%)	4 (44%)	142 (16%)	**0.024/**0.64§§ (0.07)
DRB1*0301-DQB1*0201	5 (26%)	3 (33%)	188 (21%)	0.87/1§§
DRB1*0701-DQB1*0202	6 (32%)	1 (11%)	234 (26%)	0.35/1§§

§ *P* value for Hep +TB >2 xULN vs controls,

§§ *P* value for Hep+TB >2 xULN vs Hep+TB <2 xULN.

Hep: hepatocellular type of injury, TB: total bilirubin value, OR: odds ratio.

### HLA Allele Associations with AC Hepatotoxicity Onset

To examine if specific HLA alleles potentially affect the time for liver injury onset to occur the cohort was classified into those developing DILI while on the treatment, 32 cases, and those that developed DILI after the treatment was withdrawn (delayed onset), 43 cases. The B*1801 allele was more frequently found in cases with delayed onset compared to those without (OR = 5.2; CI = 1.4–19.9, *P*/*Pc* = 0.015/0.42). In addition, the presence of A*3002 (OR = 3.8; CI = 1.7–8.7, *P*/*Pc* = 0.0011/0.021) in cases with delayed onset was significantly higher compared to those of the 885 controls. The class II allele combinations DRB1*0301-DQB1*0201 (OR = 15.0; CI = 1.8–121.1, *P*/*Pc* = 0.0026/0.07) and DRB1*1302-DQB*0604 (OR = 0.07; CI = 0.008–0.6, *P* = 0.005/0.13) were found more frequently in cases with and without delayed onset, respectively. The occurrence of DRB1*1302-DQB*0604 in cases with no delayed onset was also significantly higher when compared to controls (OR = 4.7; CI = 2.0–10.8, *P*/*Pc* = 1.8E-4/0.0049) (Table 4a). No statistically significant differences in type of liver injury distribution were seen between cases with (Hep: 40%, Chol: 37%, Mix: 23%) and without (Hep: 34%, Chol: 19%, Mix: 47%) delayed onset. Nevertheless, 61%, 73% and 40% of all hepatocellular, cholestatic and mixed cases, respectively, were seen to have delayed onset.

**Table 4:** HLA allele distribution in amoxicillin-clavulante induced liver injury patients classified by (A) presence or absence of delayed onset and (B) time to onset after drug withdrawal among delayed onset cases.

	Delayed onset n = 43	No delayed onset n = 32	*P/Pc* (OR)
A*3002	8 (19%)§	3 (9%)	0.34/1
B*1801	15 (35%)	3 (9%)	**0.015/**0.42 (5.2)
DRB1*1502-DQB1*0602	14 (33%)	8 (25%)	0.56/1
DRB1*0301-DQB1*0201	14 (33%)	1 (3%)	**0.0026/**0.07 (15.0)
DRB1*1302-DQB1*0604	1 (2%)	8 (25%)§	**0.005/**0.13 (0.07)
	≥15 days n = 11	<15 days n = 32	**P**
A*3002	2 (18%)	6 (19%)	**0.85**
B*1801	4 (36%)	11 (34%)	**0.95**
DRB1*1502-DQB1*0602	5 (45%)	9 (28%)	**0.38**
DRB1*0301-DQB1*0201	3 (27%)	11 (34%)	**0.80**
DRB1*1302-DQB1*0604	0	1 (3%)	**na**

§ *Pc* <0.05 compared to controls (n = 885), OR: odds ratio.

na: not applicable.

No significant differences in allele distribution were detected when comparing cases with 15 days or more with those having less than 15 days delayed onset. However, there was a tendency for a longer delayed onset in the presence of DRB1*1501-DQB1*0602 (Table 4b). No significant differences in the type of damage were seen between cases with more than 15 days delayed onset and those with shorter delayed onset.

### Amino Acid Analysis of HLA Alleles

The HLA alleles identified in DILI patients and controls were also analysed at amino acid level, with specific focus on the amino acids situated in the antigen binding pockets. An aspartic acid and a glycine situated at amino acids 166 and 167, respectively, in the HLA-A locus were found to reduce the risk of hepatocellular type of DILI (OR = 0.19; *P*/*Pc* = 7.01E-4/0.034), but did not have any effect on the risk of developing cholestatic/mixed type of DILI. The only amino acid found to produce a significantly enhanced risk of DILI was an arginine at residue 152 in the HLA-A locus (OR = 5.87; *P*/*Pc* = 4.75E-4/0.023) and the risk was limited to hepatocellular type of injury. No specific amino acids in the HLA loci B, C, DRB1 or DQB1 were found to provide any significant DILI risk or protection.

## Discussion

Variations in HLA allele associations in AC DILI cases have recently been demonstrated between ethnically different cohorts [8]. We hypothesised that such differences may influence phenotype presentations. In this study we found that the HLA class I alleles A*3002 and B*1801 were associated with hepatocellular injury, while the class II allele combination DRB1*1501-DQB1*0602 (known to be in strong linkage disequilibrium and subsequently form a common HLA class II haplotype) was associated with cholestatic/mixed type of liver damage. Although DRB1*1501-DQB1*0602 has previously been implicated in AC DILI, phenotype specific associations may have been overlooked in earlier studies due to smaller cohorts with a predominance of cholestatic cases [6], [7]. In contrast, 37% of the cases in our cohort presented hepatocellular damage and subsequently enabled a stratified phenotype analysis. An association between B*1801 and Spanish AC DILI patients was recently demonstrated with a clear tendency towards hepatocellular injury [8]. In addition, statistical significance was demonstrated between the presence of B*1801 and highly elevated ALT values, being suggestive of hepatocellular injury, in the same population. Similar results were seen in this study with B*1801 being significantly associated with increased ALT values but not reaching significance after Bonferroni’s correction in terms of hepatocellular type of injury despite the increase in cohort size. Hence, B*1801 is not likely to be the only risk factor for hepatocellular injury, but more likely one of several genetic factors distinguishing type of liver injury in AC hepatotoxicity. The fact that the type of liver injury is preserved in patients with multiple DILI episodes induced by different drugs and subsequently under different environmental conditions supports the idea of genetic determinants in the host controlling the injury type [15]. The ethnic restriction for the HLA-B*1801 effect as demonstrated to date highlights the importance of using ethnically correct control populations in DILI pharmacogenetic studies.

The A*3002 allele is relatively infrequent in Caucasians (phenotype distribution in our control group = 5.6%). Intriguingly, 14.7% of the DILI cases contained this allele and 72.7% of these patients manifested hepatocellular damage. In fact, the presence of A*3002 was associated with a higher risk of developing hepatocellular injury (OR = 6.7) than that of B*1801 (OR = 2.9). The risk of hepatocellular DILI remained for the A*3002-B*1801 allele combination. Both of these alleles are present in the conserved extended haplotype (CEH) HLA-A*3002-B*1801-Cw*0501-DRB1*0301-DQB1*0201, which is more frequent in southern parts of Europe. In fact, it has been shown to be the third most common CEH in Spain [16]. The elevated occurrence of A*3002 and B*1801 in Spain was evident when comparing allele frequencies in control populations from various geographic locations and may be a reason for the ethnically restricted phenotypic effects of these genes to date in AC DILI (Table S1).

Associations between DRB1*15, DQB1*06 and cholestatic/mixed type of injury was also detected in our earlier HLA genotyping study of 140 DILI patients with various causal agents (of which 17% were due to AC) [17]. One might speculate that DRB1*1501-DQB1*0602 is therefore associated with cholestatic/mixed type of damage in general and not limited to AC DILI specifically. This is supported by the reported link between DRB1*1501-DQB1*0602 and primary sclerosing cholangitis [18]. Nevertheless, liver enzyme elevations caused by lumiracoxib has recently been associated with the presence of the extended haplotype HLA-DRB1*1501-DQB1*0602-DRB5*0101-DQA1*0102 in a cohort of predominantly hepatocellular injury cases [19], which speaks in favour of DRB1*1501-DQB1*0602 having a different effect on the clinical expression in lumiracoxib DILI.

Patients over 60 years of age have previously been shown to have a higher tendency to develop cholestatic type of injury, while those younger than 60 years are more prone to suffer hepatocellular damage [3]. With this in mind the demographical findings when comparing the A*3002 and/or B*1801 carriers with those carrying the DRB1*1501-DQB1*0602 alleles also provide indirect indications of different liver injury type associations, as the cases in the class I allele group were significantly younger than those carrying the specified class II alleles. This was seen in both the mean age as well as in the proportion of patients over 60 years of age. Men were seen to predominate among the A*3002/B*1801 carriers of younger age, despite earlier findings of younger women being more prone to develop hepatocellular type of DILI [3]. A trend towards younger men being more prone to develop hepatocellular AC DILI has however been detected earlier, suggesting a more prominent role for age compared to sex. In fact, age has been shown to be the most important non-genetic determinant in the biochemical expression of AC DILI [9].

Carriers of A*3002 or B*1801 alleles were also seen to require hospitalizations more frequently than those carrying the DRB1*1501-DQB1-0602 alleles, suggesting that the presence of the two class I alleles potentially lead to a more serious nature of injury. In fact, the only two cases of the cohort that developed fulminant hepatic failure were both B*1801 carriers, with one of the patients also containing A*3002 allele.

Hyman Zimmerman observed that DILI cases with hepatocellular injury in conjunction with jaundice have at least a 10% mortality rate, now termed Hy’s Law [11]. However, the best definition of Hy’s Law cases remains a matter of debate. We found that using an R value ≥5 avoided the inclusion of cases with an important cholestatic component, which applied when using increased ALT values as inclusion criteria. The 19 cases with R ≥5 and total bilirubin >2 xULN were enriched in A*3002 and B*1801 compared to the controls. Nevertheless, the phenotypic distribution of these alleles did not differ between hepatocellular cases classified by accompanying total bilirubin value. Therefore, A*3002 and B*1801 *per se* do not seem to notably increase the risk of mortality. However, these alleles are associated with hepatocellular injury, which is the type of injury that most frequently lead to acute liver failure [13], [20]. Interestingly, the Hy’s Law cases contained a reduced proportion of DRB1*1501-DQB1*0602 carriers. This is in accordance with these alleles being associated with cholestatic/mixed type of injury, which generally has a lower mortality risk. However, the relatively small size of the cohort precluded us from establishing whether these phenotype-genotype associations could be used as predictive biomarkers for severity.

The use of cases fulfilling the Hy’s Law criteria to represent severity is not ideal as only approximately 10% of these cases are expected to have a grave outcome [13], [21]. The use of more clinically relevant end points, such as development of liver failure or death would provide more accurate data. However, our cohort only contained two patients in this category, which in fact corresponds to 10.5% of the Hy’s Law cases. It is clearly a limitation to our study that such a low number of ‘truly severe cases’ does not enable any reliable statistical analysis. Hence, further analyses using bigger cohorts of severe AC DILI cases are required to confirm these results.

A time lapse between drug cessation and DILI presentation is a common feature in AC DILI [9]. Fifty-seven percent of our cohort was seen to have delayed onset. This subgroup of patients was enriched in the HLA class I alleles A*3002 and B*1801 as well as the DRB1*0301-DQB1*0201 alleles. Surprisingly though, the proportion of hepatocellular cases was not significantly increased in this subgroup, but very similar to that of cholestatic cases. Nevertheless, the majority (61%) of all hepatocellular cases had delayed onset, which may explain the high frequency of A*3002 and B*1801. The high frequency of DRB1*0301-DQB1*0201 possibly stems from its inclusion in the same CEH as A*3002 and B*1801. Genetic variations associated with this CEH or parts thereof could play a role in determining time to onset. Transfer of non-HLA disease traits on CEH has been reported, such as susceptibility for immunoglobulin D deficiency on the aforementioned CEH, presumably located between the HLA class II and III regions [22].

Patients without delay onset were more likely to contain the DRB1*1302-DQB1*0604 allele combination. The distribution of DRB1*1302-DQB1*0604 carriers is noticeable with 89% showing no delayed onset. We are currently unable to explain the significance of this finding. One might speculate that DRB1*1302-DQB1*0604 is associated with a particularly strong immune response and subsequently mounts a faster T cell response. This has been demonstrated for HLA-DRB1*13 in other situations [23], [24]. Nonetheless, the possibility of non-HLA genes, linked to individual HLA alleles or haplotypes, being the true determinant of temporal disease initiation should not be overlooked.

The amino acids constituting the HLA molecules, especially those forming antigen binding pockets, could influence immunologic responses. We found that the presence of an arginine in position 152 of the HLA-A locus was significantly more common in the hepatocellular cases than the controls. Amino acid 152 in the HLA loci A, B and C are part of antigen binding pocket E. It is also involved in T cell receptor binding, though this function seems to be limited to the HLA-A locus. An arginine at residue 152 is present in A*3002. Though it is not specific for A*3002, its presence is mostly limited to A*30 and A*80 alleles. Arginine is also the only positively charged amino acid found at position 152 in the HLA-A alleles identified to date [25]. Hence, a positively charged residue at this position at the HLA-A locus is therefore not a requirement for AC DILI, but may enhance the risk. It is possible that an evaluation of individual amino acids focusing merely on presence or absence, while not considering information that places the residues into structural and functional context, may fail to notice essential associations and therefore does not provide a full answer.

It is interesting to note that the presence of an arginine at residue 62 in the HLA-B*1502 allele has recently demonstrated a role in carbamazepine-induced hypersensitivity producing Stevens-Johnson syndrome/toxic epidermal necrolysis. The arginine side chain is believed to form a hydrogen bond with the ketone group of 5-carboxamide of carbamazepine in the HLA-B*1502 B pocket, while an asparagine at residue 63 contributes to the specificity in molecular recognition [26]. The protective effect of aspartic acid and glycine at residues 166 and 167, respectively, in the HLA-A locus corresponds with the low number of hepatocellular DILI cases in our cohort seen to contain alleles with these specific residues: A*0101, A*2301, A*2402 and A*8001.

Though no mechanistic explanations for the detected phenotype-genotype associations can currently be offered, one can speculate that the underlying mechanism of AC DILI toxicity could involve protein-drug/metabolite complex presentation by HLA class I and II molecules and subsequent T-cell mediated cytotoxicity and cytokine production. The expression of HLA class I molecules on hepatocytes could contribute to hepatocellular injury, while HLA class II antigens have been found to be expressed on biliary epithelium, which may potentially evoke cholestatic type of injury. The role of natural killer cells, abundant in the liver and whose level of cytotoxic responsiveness is greatly influenced by HLA class I binding, in DILI warrants further studies [27].

In conclusion, our data are consistent with a role for immunological factors affecting the AC DILI signature in Spanish patients, with the HLA-A*3002 and B*1801 alleles being associated with hepatocellular pattern of injury, while HLA-DRB1*1501-DQB1*0602 increases the risk of cholestatic/mixed type of liver damage. The former correlation was reinforced in Hy’s Law cases and the two HLA class I alleles were also more prominent in cases with delayed onset. In contrast, DRB1*1302-DQB1*0604 carriers were scarcely seen to have delayed onset. The findings highlight a role for genetics, not only in AC DILI susceptibility, but also in phenotype expression.

## Supporting Information

Table S1 HLA class I and II allele frequencies in the current study compared to different populations. The allele frequencies were obtained from the Allele frequency net database^1^.
(DOC)
