# Correction: HLA Alleles Influence the Clinical Signature of Amoxicillin-Clavulanate Hepatotoxicity

**DOI:** 10.1371/journal.pone.0112165

**Published:** 2014-10-23

**Authors:** The PLOS ONE Staff

The following information is missing from the Funding section: This study was supported by grant EU-ERDF (PI12/00378) from the Instituto de Salud Carlos III.
