# Supplementary material for: HLA Alleles Influence the Clinical Signature of Amoxicillin-Clavulanate Hepatotoxicity
Source: PLoS One. 2013 Jul 9;8(7):e68111. doi: 10.1371/journal.pone.0068111 (PMC3706603; doi:10.1371/journal.pone.0068111)
Supplement: Table S1 — HLA class I and II allele frequencies in the current study compared to different populations. The allele frequencies were obtained from the Allele frequency net database1. (DOC) [file pone.0068111.s001.doc]

**Table S1.** HLA class I and II allele frequencies in the current study compared to different populations. The allele frequencies were obtained from the Allele frequency net database^1^.

|  | **Allele frequency** | | | | | |
| --- | --- | --- | --- | --- | --- | --- |
| **HLA allele** | **Current study** n = 885 | **Southern Spain** n = 99 | **Germany** n = 8862 | **USA** n = 1070 | **Morocco** n = 98 | **China** n = 618 |
| ***Class I*** |  |  |  |  |  |  |
| A*30 | 0.056 | 0.045 |  | 0.004 | 0.041 |  |
| A*3002 | 0.029 |  | 0.006 |  |  | 0.004 |
| B*18 | 0.096 | 0.088 |  | 0.041 | 0.041 |  |
| B*1801 | 0.096 |  | 0.047 |  |  | 0.004 |
| ***Class II*** |  |  |  |  |  |  |
| DRB1*0301 | 0.120 | 0.131 | 0.109 | 0.104 | 0.117 | 0.035 |
| DRB1*0701 | 0.170 | 0.177 | 0.121 | 0.126 | 0.163 | 0.095 |
| DRB1*1302 | 0.046 | 0.056 | 0.042 | 0.052 | 0.056 | 0.023 |
| DRB1*1501 | 0.093 | 0.086 | 0.145 | 0.139 | 0.128 | 0.076 |
| DQB1*0201 | 0.122 | 0.126 |  |  | 0.1230 |  |
| DQB1*0202 | 0.146 | 0.177 |  |  | 0.197 |  |
| DQB1*0602 | 0.089 | 0.091 |  |  | 0.129 |  |
| DQB1*0604 | 0.034 | 0.040 |  |  | 0.018 |  |

^1^Gonzalez-Galarza FF, Christmas S, Middleton D and Jones AR. (2011). Allele frequency net: a database and online repository for immune gene frequencies in worldwide populations. Nucleic Acids Res 39: D913-D919. ([www.allelefrequencies.net](http://www.allelefrequencies.net/) accessed April 2013)
